# A modular microfluidic system based on a multilayered configuration to generate large-scale perfusable microvascular networks

**DOI:** 10.1038/s41378-020-00229-8

**Published:** 2021-01-06

**Authors:** Tao Yue, Da Zhao, Duc T. T. Phan, Xiaolin Wang, Joshua Jonghyun Park, Zayn Biviji, Christopher C. W. Hughes, Abraham P. Lee

**Affiliations:** 1 Department of Biomedical Engineering, University of California, Irvine, CA USA; 2 School of Mechatronic Engineering and Automation, Shanghai University, Shanghai, China; 3 Department of Molecular Biology and Biochemistry, University of California, Irvine, CA USA; 4 Department of Micro/Nano Electronics, Shanghai Jiao Tong University, Shanghai, China; 5 National Key Laboratory of Science and Technology for Micro/Nano Fabrication, Shanghai Jiao Tong University, Shanghai, China; 6 Key Laboratory for Thin Film and Micro Fabrication of the Ministry of Education, Shanghai Jiao Tong University, Shanghai, China; 7 Department of Electrical Engineering and Computer Science, University of California, Irvine, CA USA; 8 Department of Applied Mathematics - Biology, Brown University, Providence, RI USA; 9 Department of Mechanical and Aerospace Engineering, University of California, Irvine, CA USA

**Keywords:** Engineering, Microfluidics

## Abstract

The vascular network of the circulatory system plays a vital role in maintaining homeostasis in the human body. In this paper, a novel modular microfluidic system with a vertical two-layered configuration is developed to generate large-scale perfused microvascular networks in vitro. The two-layer polydimethylsiloxane (PDMS) configuration allows the tissue chambers and medium channels not only to be designed and fabricated independently but also to be aligned and bonded accordingly. This method can produce a modular microfluidic system that has high flexibility and scalability to design an integrated platform with multiple perfused vascularized tissues with high densities. The medium channel was designed with a rhombic shape and fabricated to be semiclosed to form a capillary burst valve in the vertical direction, serving as the interface between the medium channels and tissue chambers. Angiogenesis and anastomosis at the vertical interface were successfully achieved by using different combinations of tissue chambers and medium channels. Various large-scale microvascular networks were generated and quantified in terms of vessel length and density. Minimal leakage of the perfused 70-kDa FITC-dextran confirmed the lumenization of the microvascular networks and the formation of tight vertical interconnections between the microvascular networks and medium channels in different structural layers. This platform enables the culturing of interconnected, large-scale perfused vascularized tissue networks with high density and scalability for a wide range of multiorgan-on-a-chip applications, including basic biological studies and drug screening.

## Introduction

The human blood circulatory system comprises a closed network of vessels that allow blood to circulate throughout the body for gas exchange and mass transport. which is essential to maintaining organ viability. To mimic the characteristics and functions of human organs in vitro, it is necessary to integrate fully functional microvascular networks for a more physiologically accurate organ-on-a-chip^1^. A microvascular network is necessary to build large, 3D, and fully functional tissues in vitro, since the tissues need to be no more than 200 μm away from a capillary to obtain nutrients through diffusive transport^2^. Microvascular networks play an important role in maintaining the biological functions of tissues, including the delivery of nutrients and drugs from capillaries as well as the removal of wastes from surrounding tissues^3^. Therefore, to better study these processes and understand the underlying biological mechanisms, large-scale and perfusable in vitro human microvascular networks are needed.

There are several approaches to generate in vitro large and small vascular networks, including microfluidic device-based methods, such as microfabrication and manipulation^4–7^, hybrid methods, such as EC lining of microfluidic channels^8–11^, scaffold-based methods^12–15^, and biology-driven methods, such as de novo flow-induced vascular formation inside microfluidic devices^16–20^. Previously, our perfused microvascular networks were developed by engineering anastomoses between microvascular capillary networks and microfluidic channels^16^. Unlike other methods, this platform provides a 3D microenvironment that is suitable for cell growth and the formation of microvascular networks via vasculogenesis. Cells are not forced to coat vascular-like structures in a microfluidic device to form artificial vessels but are induced to do so in the native microenvironment (flow, chemical gradients, etc.) controlled by the microfluidic device. This platform has proven successful in generating consistently perfusable vascularized tissues that have already been utilized for drug screening applications^21^.

However, in our previous work, the tissue chambers and culture medium channels were all contained within one device layer. This single-layer configuration provides limited space for both the tissues and the culture medium channels, which results in a crowded layout and limited design options to position the microvascular networks^21^. It also limits the flexibility to design multiple interconnected vascularized micro organs (VMO) on the same platform.

Potential solutions to overcome these problems stem from the use of modular microfluidic systems, in which different components are separated and individually designed and fabricated^22–24^. Here, we propose a modular approach with a multilayered configuration that separates the tissue chambers and culture medium channels into different device layers^25^, where both layers can be fabricated and modified individually. This allows the tissue chambers to be densely packed, while the supplied culture medium can flow vertically into multiple tissues. More importantly, the two device layers can be combined freely, forming various flow conditions inside an array of tissue chambers. As interstitial flow is a key factor in regulating capillary growth^26^, this platform can be used to create a wide range of flow conditions to study vasculogenesis and angiogenesis.

In this paper, we report a two-layer PDMS modular microfluidic system and demonstrate its use in generating large-scale microvascular networks that cover more than 14 mm^2^, with a vessel density of 12 mm/mm^2^. To prevent the hydrogels from leaking into the culture medium channels during gel loading into the tissue chambers, a novel plastic mold-based fabrication method was developed to generate semiclosed rhombic medium channels. Complex microvasculature with varying morphology was grown using this two-layer modular microfluidic system. Vertical vascular anastomosis between the two device layers was demonstrated, and 70-kDa FITC-dextran perfusion could be performed without leakage in both the anastomotic connections and the microvascular networks, demonstrating the strong barrier property of the vasculature lumens and the tight interconnection between the culture medium channels and the vascular capillary network underneath. Ultimately, this flexible and scalable modular platform is able to accommodate multiple organs on a chip interconnected via the perfused microvasculature, forming the basis of a functioning “body-on-a-chip”.

## Materials and methods

### Modular microfluidic system based on multilayered configurations

**Fig. 1:**
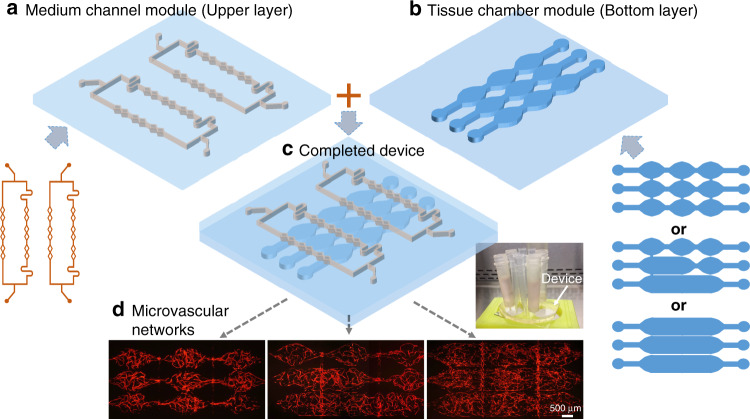
The modular microfluidic system combining two PDMS layers. **a** Medium channel module (upper layer) and **b** tissue chamber module (bottom layer). Different channel designs are used for these two modules. **c** A completed two-layered device shows the medium channels and tissue chambers in different layers. **d** Large-scale perfused microvascular networks generated by using different configurations

Microvascular networks were generated in a diamond-shaped tissue chamber in our previous works^16,19^. However, in this configuration, the culture medium channels and tissue chambers were in the same PDMS layer, limiting the space and layout flexibility for the channels and tissues, as well as the range of fluidic conditions, used to induce vascular formation. Here, a modular microfluidic system using two PDMS layers is developed, enabling large-scale, interconnected, multichamber microvascular networks with spatial flow control. In this approach, the channels used to deliver culture medium and the tissue chambers are fabricated separately in two PDMS layers. As shown in Fig. 1a–c, the tissue chambers and culture medium channels are aligned and bonded to form functional devices with various channel configurations. The medium channels in the upper layer were designed with 4 lines to supply the culture medium to the tissue chambers underneath. For the tissue chambers in the bottom layer, the previous basic design of the diamond shape was modified and designed with three types of tissue chambers, including diamond, half-rectangle, and rectangle shapes, to form various configurations. Because different upper and bottom layers can be combined with various degrees of freedom, and different channel-chamber combinations can be used to form various fluidic flow profiles to induce vascular formation, this system constitutes a modular vascularized micro organ system.

### PDMS device design and fabrication

**Fig. 2:**
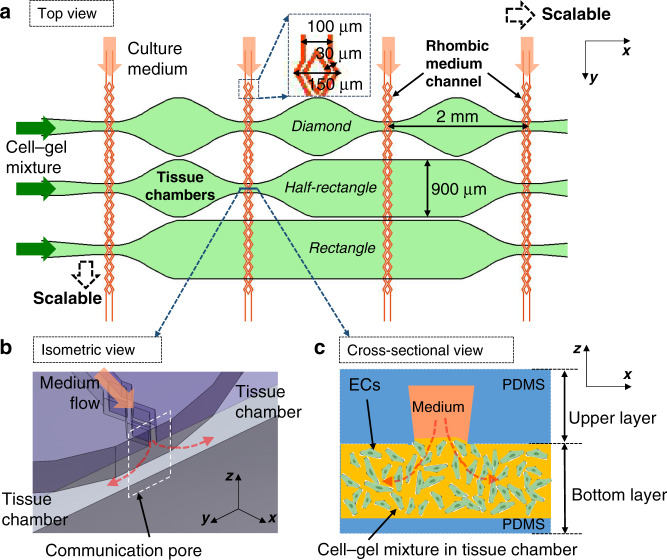
Schematic drawing of an example of a device and the simulated flow conditions inside. **a** Top view of the device layout with a 3 × 3 tissue matrix. Cell-embedded fibrin gels were loaded into the bottom tissue chambers. Then, the culture medium was supplied through the upper medium channels. **b** The isometric view of a communication pore shows how the medium flows from the medium channel to the tissue chamber. **c** The cross-sectional view of the communication pore shows how endothelial cells (ECs) form lumens connecting the medium channel and the tissue chamber

Since various combinations of upper and bottom layers can be used in our system, one combination is shown here in Fig. 2 as an example to describe the concept of the device design and fabrication. In the upper layer, the straight part of the culture medium channel has a 100-μm width, and its rhombic part has a 150-μm width. The bifurcated channels in the rhombic part have a 30-μm width. In the bottom layer, the tissue chamber array consists of three types of tissue chambers, including chambers with a diamond, a half-rectangle and a rectangle shape. The diamond-shaped chambers are similar in design to chambers used in our previous works^16,19^.

For each device, the upper layer and bottom layer were both made of PDMS (Sylgard-184, Dow) cast from SU-8 patterned silicon wafers using standard soft lithography techniques^27^. The heights of the microfluidic channels and the tissue chambers were all 100 μm. One upper layer and one bottom layer were aligned as shown in Fig. 2a under the microscope and bonded by plasma bonding. The culture medium channels in the upper layer and the tissue chambers in the bottom layer were overlaid after bonding. The medium-tissue overlay areas (hereafter denoted as the “overlaid areas”) form an opening space connecting the medium channels and the tissue chambers, which are called communication pores, as shown in Fig. 2b, c. After plasma bonding, bottomless plastic vials serving as culture medium reservoirs were glued to the top of the inlets and outlets of the medium channels. The fabricated devices were autoclaved at 121 °C for 30 min before use in subsequent experiments.

### Rhombic medium channel design and fabrication

**Fig. 3:**
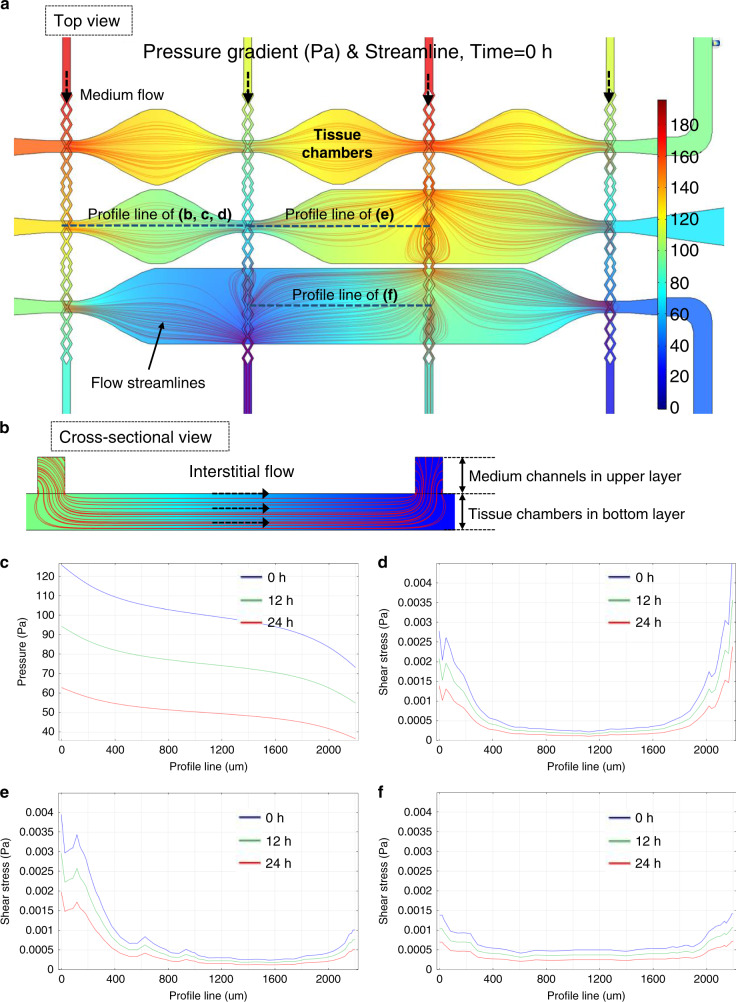
Numerical simulations of flow inside the tissue chambers. **a** The pressure gradient and flow stream of 3 types of chambers, including diamond, half-rectangle, and rectangle chambers. **b** The culture medium diffused from the upper medium channel into the tissue chamber, generating interstitial flow inside. **c** The pressure drop across one tissue chamber was ~50 Pa, which was sufficient for stimulating vessel growth. The shear stress distribution inside the **d** diamond, **e** half-rectangle and **f** rectangle-shaped tissue chambers were different, which provided various flow conditions to induce the formation of larger and denser microvascular networks inside

**Fig. 4:**
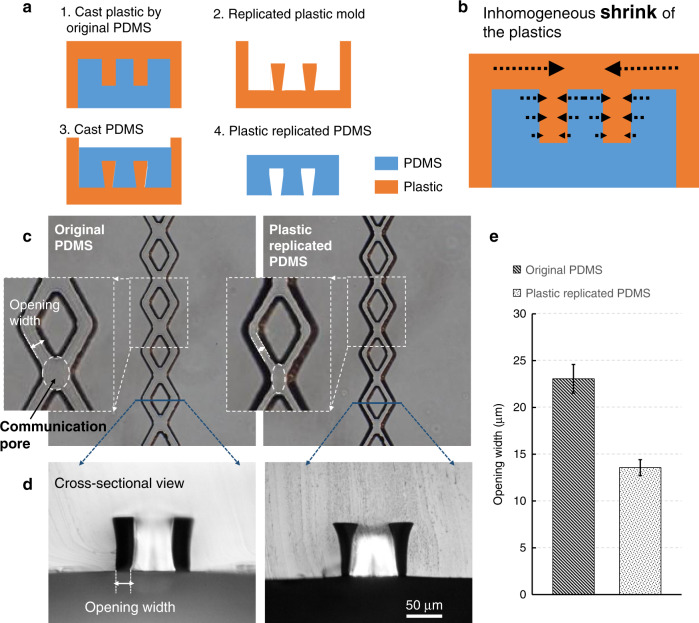
Semiclosed rhombic medium channels were fabricated using plastic molds. **a** Fabrication procedures for replicated plastic PDMS with semiclosed rhombic medium channels. **b** The inhomogeneous shrinkage mechanism causes the PDMS channel to remain semiclosed. **c** The images of the original PDMS and replicated plastic PDMS show the size decrease of the opening width and the communication pores. **d** The cross-sectional view clearly shows the semiclosed medium channels. **e** The quantitative data demonstrate that the opening width can be reduced to be approximately half of that of the original channels. Mean ± SD, *n* = 8

To provide sufficient culture medium and prevent gel bursting, a rhombic medium channel was designed and fabricated, as shown in Figs. 3a and 4c. The width of the opening of the bifurcated rhombic channels was 30 μm. The PDMS layer of the rhombic medium channels was fabricated as shown in Fig. 4a. After the original PDMS layer was replicated from the SU-8 wafers, it was then used as a master mold to cast a liquid plastic replica (customized polyurethane, Smooth Cast 310, Smooth-On, Inc). After 24 h, the solidified plastic replica was demolded. The microfluidic feature dimensions were slightly reduced in this plastic mold, since the liquid plastic underwent a volumetric change during the curing process, resulting in a linear dimensional shrinkage (~4%, as mentioned in ref. ^28^) compared to the original SU-8 mold. The final PDMS layer containing the rhombic medium channels was cast using this replicated plastic mold.

### Cell culture

Human endothelial colony forming cell-derived endothelial cells (ECFC-ECs) were isolated from cord blood as previously described^29^ and expanded in gelatin-coated (0.5%) flasks in EGM-2 medium (Lonza). ECs were transduced with lentivirus constructs (Addgene) to express mCherry fluorescent protein and used once they reached passage 4–7. Normal human lung fibroblasts (NHLFs) were purchased from Lonza, cultured in DMEM (Corning) containing 10% fetal bovine serum (FBS, Gemini Bio Products), and used once they reached passage 4–7. All cell types were grown in a 37 °C/5% CO_2_/20% O_2_ incubator in 100% humidified air before use in experiments. Here, the fibroblasts served as pericytes in the coculture system. Fibroblasts can stabilize and promote the maturation of networks in microfluidic devices. The soluble protein secreted by fibroblasts supported not only EC sprouting but also lumen formation of the vascular networks. Fibroblast-derived proteins also increased the stiffness of the ECM, thus partially inducing lumen formation by ECs. Furthermore, fibroblasts can secrete a variety of angiogenic growth factors during tissue growth^30,31^.

### Cell loading procedures and on-chip culture

The cell loading procedures included cell-gel mixture preparation, cell-gel mixture loading into the devices, medium channel coating and culture medium loading. The whole process must be completed within 90 min. First, the cell-gel mixture was prepared by suspending ECs (5 × 10^6^ cells per mL) and NHLFs (5 × 10^6^ cells per mL) in fibrinogen solution (10 mg mL^−1^, Sigma-Aldrich)^16,19,32,33^. Second, the suspension was mixed with 50 U mL^−1^ thrombin (Sigma-Aldrich) at a final concentration of 3 U mL^−1^ and quickly injected (less than 10 s) into the tissue chambers through the gel loading port (green arrows in Fig. 2a) using a micropipettor. The gel was allowed to polymerize in an incubator at 37 °C for 15 min. Third, laminin (1 mg mL^−1^, Life Technologies) was introduced through the medium loading port (red arrows in Fig. 2a) to coat the inner surface of the medium channels. Finally, EGM-2 (Lonza) culture medium was supplied through the same culture medium channels (red arrows in Fig. 2a) under a relatively stable pressure decrease from the inlets to the outlets.

The device was perfused with culture medium driven by hydrostatic pressure. As shown in Fig. S1, the culture medium was at different heights in the four reservoir vials to generate hydrostatic pressure. Initially, the medium level on one side of both the rhombic medium channels was the same and was 10 mm higher than that on the other side. During medium perfusion over ~48 h, the medium level on both sides gradually equalized. Then, the cell culture medium level in the vials was adjusted to the original 10 mm difference in height between both sides, while the fluidic direction in the medium channels was reversed by switching the sides with high and low hydrostatic pressure. The medium refill procedure was conducted every 48 h to induce vascular formation. The whole microvascular network was fully developed after ~10 to 12 days in the device. Then, the networks were perfused with culture medium for approximately another 14 days, which was limited by the proliferation capability of the primary ECs as well as the degradation of the fibrin hydrogels.

### Dextran-FITC perfusion

To validate the anastomosis of the microvascular networks and to evaluate vascular perfusivity, a dextran-FITC perfusion test was conducted. The medium was first replaced by DPBS with a similar hydrostatic pressure profile, and the device was placed onto a microscope stage. Then, 70 kDa-FITC dextran (final concentration of 50 μg mL^−1^, Sigma-Aldrich) was added to the medium reservoir on the side with high hydrostatic pressure, which allowed the 70 kDa-FITC dextran to perfuse through the culture medium channels into the microvascular networks. A time-lapsed image sequence was recorded for 10 min to capture the flow of 70 kDa-FITC dextran in the device.

### Data acquisition and analysis

Bright-field and fluorescent images were obtained using an optical microscope (Olympus IX51 and Canon 5D II camera). Confocal images were obtained using a confocal laser scanning microscope (A1Rsi-N, Nikon). Images of the capillaries were processed by ImageJ (Ver. 1.47, NIH) and AngioTool (Ver. 0.6a, NIH) to calculate the total vessel length, total number of junctions, and average vessel length. Data are shown as the mean ± standard deviation (SD) unless stated. Estimated means and standard deviations were calculated using Microsoft Excel.

## Results

### The modular microfluidic system provides various flow conditions in tissue chambers

The presented modular microfluidic system based on a two-layered configuration can provide freedom of design to combine and connect a large number of tissue chambers. As previously demonstrated, ECs are responsive to interstitial flow during vasculogenesis, and interstitial flow can be controlled spatially to form microvascular networks with different mass transport configurations^33,34^. It is possible to regulate the vessel growth pattern and obtain large-scale microvascular networks in our presented modular microfluidic system by optimizing the interstitial flow profiles. Therefore, we first investigated the interstitial flow conditions, such as the pressure gradient and shear stress distribution, in different tissue chambers. Numerical fluidic simulations were conducted using COMSOL Multiphysics (Ver. 5.2a, Comsol AB, Stockholm, Sweden). The numerical values used in these simulations, including the material properties of fibrinogen hydrogels and the hydrostatic pressure profile used to generate fluidic flow, were based on our previous works^16,21,35^.

Figure 3a shows an example of these simulation results. In this simulation, fluid flow is driven by hydrostatic pressure inside the microfluidic channels, and fibrinogen hydrogels are placed inside the tissue chambers. The tissue chambers have three different designs, diamond-, half-rectangle- and rectangle-shaped, to investigate how different tissue chamber shapes influence the interstitial flow profile inside. The rainbow color scheme represents the pressure gradient, and the red lines show the flow stream. By drawing profile lines across these chambers, quantitative analysis of the flow conditions can be performed. Because fibrinogen hydrogel is a porous material, the interstitial flow of culture medium through it was simulated, as shown in Fig. 3b. The culture medium was perfused from the upper layer medium channels to generate interstitial flow across all lower tissue chambers, which provided stimuli for microvascular growth.

As shown in Fig. 3c, the pressure drop from the left end to the right end of one chamber was approximately 50 Pa at different time points during one full culture cycle. Based on our previous data^16^, this two-layered configuration provided a sufficient pressure gradient to stimulate vessel growth. Different tissue chamber designs were compared to estimate the shear stress distribution, a key factor that regulates vascular development. The hypothesis is that shear stress distribution will determine the growth and connectivity of the microvascular networks inside the chambers, especially in terms of vessel length and density^26,36^. The shear stress distribution inside the diamond-, half-rectangle- and rectangle-shaped tissue chambers are shown in Fig. 3d–f, respectively. Although the lowest shear stresses in these three chamber designs are almost the same, the rectangle-shaped chamber has the lowest average shear stress and a more even distribution. Here, we chose the configuration with three different shapes in one device as an example to calculate different interstitial flow profiles. In the real experiments, any layout can be used as long as it can be aligned to the culture medium channels, as shown in Fig. S2. By using different shapes for the tissue chambers, various interstitial flow profiles are generated in this modular microfluidic system, which is an ideal platform to optimize the conditions required to generate larger and denser microvascular networks.

### Semiclosed rhombic medium channels prevent gel bursting during cell loading

Based on our design concept and numerical simulations, the two-layered configuration provides a flexible design to spatially pattern the culture medium channels relative to the tissue chambers. However, two challenges were posed in practice. First, we had to ensure that the cell-gel mixture would not burst into the upper layer medium channels and clog them during the loading process. Second, the culture medium needs to be easily introduced into the medium channels and perfused into the hydrogel underneath.

As shown in Fig. 2c, there is an opening between the medium channels and the cell-gel mixture in the tissue chambers. This opening space has to be large enough to provide sufficient culture medium for cell growth. However, too large of this opening may cause gel bursting into the medium channels during the cell loading process^35^. Therefore, to prevent gel bursting, pillars were first utilized, as shown in Fig. S3. There was no gel bursting in this case, but when culture medium was introduced into the medium channels, air bubbles were easily trapped by the pillars because of the hydrophobic surface of PDMS. The bubbles blocked the culture medium from flowing into the hydrogel. Therefore, a novel rhombic-shaped medium channel was employed. Because the 2 divided channels cross every 100 μm in this design, bubbles can be easily flushed out of the channels during the loading of culture medium. Experiments demonstrated that this rhombic medium channel design is exceptionally good at preventing air bubbles, as shown in Supplementary Materials Video S1.

The crossing sections of the rhombic medium channels act as communication pores, as shown in Fig. 4c. As these pores are relatively wider than the divided rhombic channels, gel bursting is more likely to take place at these pores. To prevent gel bursting while preserving the rhombic shape to avoid air bubbles, the dimensions of these pores had to be reduced during fabrication. However, after reducing the size of the communication pores, the rhombic channels were too narrow to allow enough flow of medium for cell growth. Therefore, the ideal solution was to reduce the width of the bottom opening to make it semiclosed but not to uniformly reduce the width of the top of the diverged rhombic channels. However, such nonuniform features along the vertical direction are quite difficult to achieve by conventional standard soft lithography techniques. In this case, a type of semiclosed rhombic medium channel was generated based on our novel fabrication approach using plastic molds. Figure 4a shows our plastic replication method, which was used to reduce the opening width. Because the plastic will shrink at a rate of 4% in linear dimensional size during solidification^28^, the feature dimensions of these plastic molds are slightly different compared to those of the original SU-8 mold.

In theory, the shrinkage ratio of plastics is isotropic. However, bulk plastics shrink more in terms of actual distance during shaping of the plastic mold, as shown in Fig. 4a2. Due to the geometry of the microfluidic channels that resulted in a nonisotropic shrinkage in the horizontal direction, the plastic mold cast from the original PDMS was not isotropic, as shown in Fig. 4b. Therefore, the opening widths in the final PDMS channels replicated from these plastic molds were smaller than those cast from the original SU-8 mold, as shown in Fig. 4c. As a result, the communication pores decreased in size, but the widths of the tops of the rhombic channels were unchanged. The cross-sectional images of the channels in Fig. 4d show the narrow opening of the medium channels. As shown in Fig. 4e, quantitative analysis of the data demonstrated that the opening width could be reduced to approximately half of that of the original channels. This fabrication method is very simple and can modify the channel shape in a nonisotropic fashion.

### Vertical anastomosis at the communication pores connects the microvascular networks and the medium channels

**Fig. 5:**
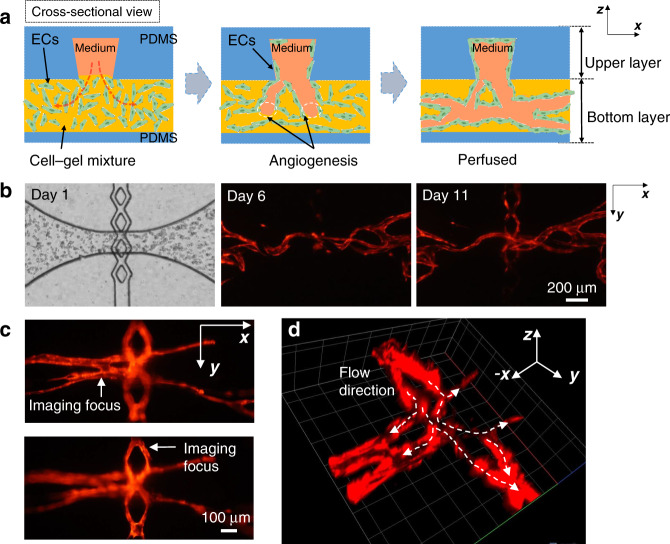
ECs grew vertically at communication pores to form a leakage-free seal between the microvascular networks and medium channels. **a** Schematic drawings of vertical anastomosis at the communication pores show that the ECs sealed the inner walls and formed connections. **b** Time-lapsed images clearly show that the ECs grew at the communication pores and formed connections. **c** Different images demonstrating the connections between the upper and lower layers. **d** Confocal images showing the full connections and how the culture medium flows though these connections into the capillaries

After the cell loading process, the devices were cultured for another 10–11 days to allow the ECs to proliferate and form lumenized microvascular networks inside the tissue chambers. Unlike those in the single-layer device, the ECs near the communication pores in the bottom tissue chambers grew vertically upward into the medium channels in the upper layer, sealing the inner walls and forming connections between the medium channels and the tissue chambers, as illustrated in the schematic drawings in Fig. 5a. This is due to the medium flowing from the medium channels into the bottom tissue chambers and creating a growth factor gradient that stimulates EC migration. This vertical migration eventually connects the microvascular networks and the medium channels, forming leakage-free seals at the communication pores, which we called ‘vertical anastomosis’. The time-lapsed images in Fig. 5b clearly show ECs migrating towards the communication pores, resulting in vertical anastomosis.

Because the medium channels and tissue chambers are located in two separate PDMS layers at different positions on the *z*-axis, they cannot be viewed together in the same microscope focal view plane. However, changing the focal view planes can confirm the vertical anastomosis of ECs at the communication pores. As shown in Fig. 5c, the focal view planes of the vessel segments in the medium channels and those in the tissue chambers were approximately 100 μm apart in the *z*-direction, which is in line with the design of the 200-μm combined thickness of both the medium channel and the tissue chamber. Confocal images were obtained to better visualize the complex connections between the microvascular networks and the vessel segments that lined the medium channels (Supplementary Materials Video S2). These images confirmed anastomosis at the communication pores. As shown in Fig. 5d, the microvascular networks inside the tissue chambers were fully connected to the medium channels in the upper layer.

### Large-scale microvascular networks were generated in the modular microfluidic system

**Fig. 6:**
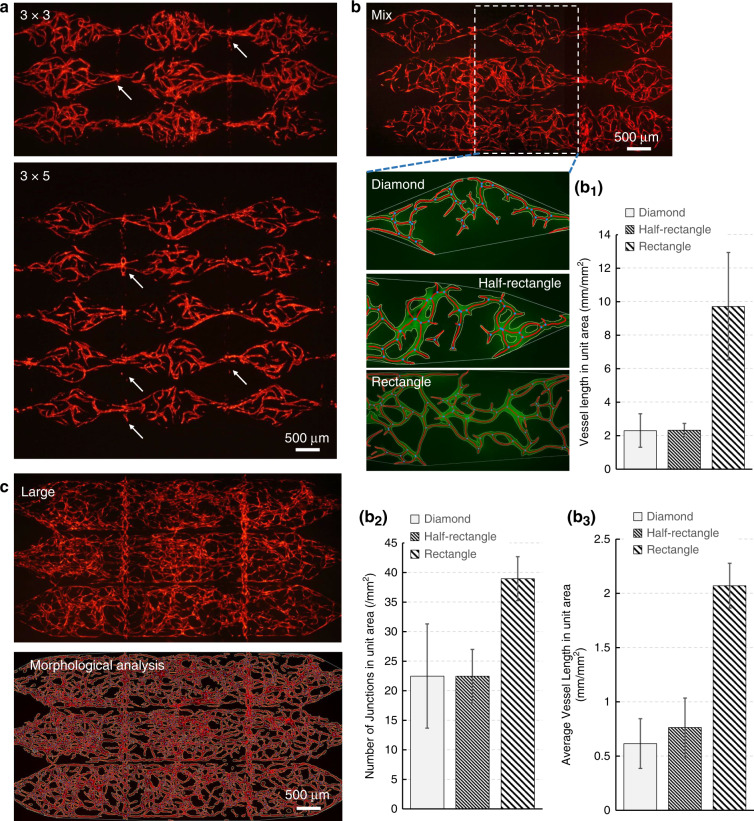
Various microvascular networks are generated in the modular microfluidic system. **a** The scalability was demonstrated by expanding the tissue chamber matrices from 3 × 3 to 3 × 5. **b** The different morphological properties of the capillaries generated using diamond-, half-rectangle- and rectangle-shaped chambers were analyzed. The rectangle-shaped tissue chambers generated the largest capillaries. **c** A large-scale microvascular network covering more than 14 mm^2^ with a vessel density of 12 mm/mm^2^ was generated by the tissue chamber containing three rows of rectangle chambers. Mean ± SD, *n* = 6

As a modular microfluidic system, one advantage of the system developed in this study is its scalability. We tested two different layouts of the tissue chamber with 3-by-3 and 3-by-5 matrices to demonstrate the scalability of our system. As shown in Fig. 6a, microvascular networks were successfully generated using both the 3-by-3 and 3-by-5 chamber matrices. Furthermore, vertical anastomosis was observed in all cases. While scaling up from a 3-by-3 to a 3-by-5 configuration may not seem significant, we used this example to demonstrate the feasibility and scalability of our modular microfluidic system to larger tissue chamber matrices of *N* by *N*.

Another advantage of this modular system is its flexibility, especially in terms of its capacity to generate various fluidic conditions by using different designs of the tissue chambers. These fluidic conditions could guide the spatial patterning of larger and denser microvascular networks. A tissue chamber design containing three shapes was utilized to investigate how different fluidic conditions influence vascular formation. As shown in Fig. 6b, diamond, half-rectangle, and rectangle chamber shapes with area sizes of 1.1 mm^2,^ 1.7 mm^2^, and 2.1 mm^2^, respectively, were used to generate tissues with different geometries. FITC-dextran (70 kDa) was perfused to quantitatively analyze the vessel morphology. AngioTool (National Cancer Institute) software was used to calculate the vessel length, number of junctions and average vessel length per unit area (mm^2^), which together represent the morphological properties of the microvasculature. The quantifications (Fig. 6b) showed that the rectangle-shaped vascularized tissue had the highest values for all three parameters, identifying the rectangle-shaped tissue chambers as the optimal configuration to generate large-scale microvascular networks. This confirms the fluidic numerical simulation results of the three types of chambers shown in Fig. 3, indicating that lower levels of shear stress promote the formation of longer and denser vessels^26,36,37^.

Figure 6c illustrates large-scale microvascular networks in three rows of rectangle-shaped tissue chambers. The microvascular networks robustly developed in the whole tissue chamber array and formed anastomotic connections with the rhombic channels in the upper layer at the communication pores. Quantitative morphological analysis was conducted, and the entirety of the microvascular networks covered more than 14 mm^2^, with a vessel density of 12 mm/mm^2^.

### Fluorescent dextran perfusion demonstrates that the microvascular networks and the medium channels are tightly connected

**Fig. 7:**
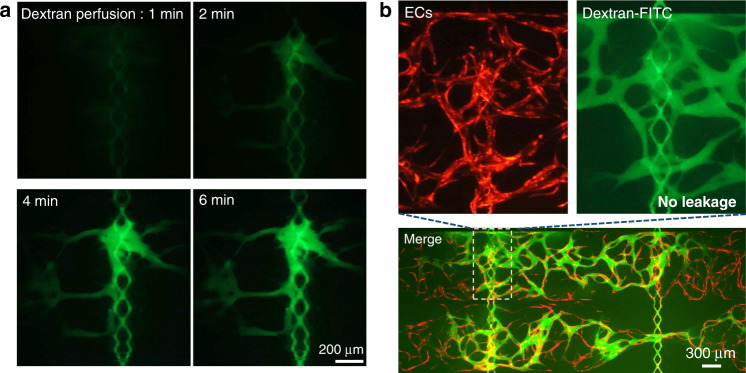
70-kDa FITC-dextran perfusion demonstrated the tight interconnections between the microvascular networks and the medium channels. **a** Time-lapsed images of FITC-dextran perfusion from medium channels into capillaries show the connectivity and perfusability. **b** The whole perfused microvascular network demonstrated the strong barrier property of the microvascular networks and the tightness of the anastomosis

To validate the integrity of the microvascular networks and the occurrence of vertical anastomosis at the communication pores, 70-kDa FITC-dextran was perfused throughout the whole device. Dextran (70 kDa) was chosen since it has a molecular weight close to that of human serum albumin (MW ∼66.5 kDa), the main protein in human plasma, making it suitable for testing endothelial barrier function. Figure 7a shows time-lapsed images of FITC-dextran perfusion from the medium channels into the microvascular network. After FITC-dextran was loaded into the medium inlets, a fluorescence microscope was used to continuously monitor dye flow at the communication pores. Time-lapsed recording was started when green fluorescence appeared in the medium channels. Within 2 min, FITC-dextran began to be perfused into the microvascular network through the communication pores. Over time, the fluorescence intensity in the vessel lumen increased due to increasing dextran influx. After 6 min, FITC-dextran has passed through the vessels near the communication pores and filled the entire microvascular network inside the tissue chambers underneath. This demonstrated the connectivity and perfusability of the microvascular networks. Furthermore, no FITC-dextran was observed outside of the vessels during the whole perfusion test, including in the communication pores where ECs formed the vertical anastomosis. As shown in Fig. 7b, after ~15 to 20 min, a whole microvascular network was filled with FITC-dextran, and there was no obvious nonphysiological leakage of FITC-dextran, especially within the entirely of the communication pore area.

For different chamber layouts (diamond, half-rectangle, and rectangle), the areas overlaid with the upper medium channels are different and require different numbers of ECs to form a conforming coating for leakage-free anastomosis. On the other hand, a larger overlaid area results in a greater number of communication pores, through which ECs can migrate into the medium channels and endothelialize the area. As shown in Fig. 6a–c, after the same culture time (11 days), there were more endothelialized channels (rhombic medium channels showing red florescence) in the device with rectangle-shaped chambers than in that with diamond-shaped chambers, which means that the devices with different chamber layouts were endothelialized and were ready to be used in a similar time frame. In conclusion, leak-free sealing at the communication pores resulting from the tight interconnections between the microvascular networks and the medium channels was confirmed. Additionally, the strong endothelial barrier of the microvascular networks and the tightness of the anastomosis have been demonstrated.

## Discussion

The presented microfluidic system is intrinsically modular, as different patterns of medium channels in the upper layer and different patterns of tissue chambers in the bottom layer can be independently utilized. Although the medium channels presented in this paper are coupled (2 medium channels have the same inlet), decoupling of channels with separate inlets can provide more flexibility to control the pressure gradients inside the tissue chambers. The tissue chambers on the bottom layer can also be flexibly designed. In previous designs, it was difficult to introduce cells or chemical compounds into the extracellular matrix (ECM) after microvascular networks had formed since there were no secondary channels connected to the ECM. However, with this two-layer configuration, adding more channels to the bottom layer to provide additional routes to introduce cells afterward or to collect intervascular efflux for quantitative analysis becomes feasible. Another interesting idea is to connect multiple rows of tissue chambers that are separated into single-layer configurations. Using the vertical anastomosis configuration described here, direct communication between each tissue row is made feasible. If different cell types are loaded into different rows, communication between the different tissue chambers can be realized through the microvascular networks in between or the microfluidic channels above the chambers (representing arterial or venule vessels). Not only can multiple different tissues be connected on this platform, the same tissues (organs) can be placed in an array on the same layer to deliver drugs into one chamber to determine how this delivery is coupled with and impacts other tissues connected to the chamber. For example, one can have “healthy” organs in one chamber and deliver therapeutic drugs to “diseased” organs in another chamber to evaluate side effects. Since immune responses usually require cell migration, one could create a scenario in which one could observe immune cells in one chamber responding to infarction or lesions in another chamber. Another example involves creating tissue chambers with different endothelial cell densities to see how they respond to various treatments. Since different organs have different vascular densities, one can utilize this platform to recapitulate the organ vasculature physiology. Ultimately, this platform will enable the development of a more complex and physiologically relevant multiorgan-on-a-chip or ‘body-on-a-chip’ system to study the interactions between different organs or to validate the circulatory functions of these microvascular networks. Beyond the two-layered device presented in this work, a multilayered configuration with more than two layers could provide higher throughput and the possibility of using thicker tissues to study larger viable vascularized tissues that more closely approach the in vivo dimensions.

To create the vertical two-layer platform, a plastic mold fabrication method was developed to provide microchannels with a sufficiently narrow width in the direction of the tissue chamber, which forms a capillary burst valve to help prevent gel bursting during the loading process. Additionally, by leveraging inhomogeneous shrinkage due to the channel geometry, this plastic replication-based fabrication approach can generate features along the vertical direction to obtain special microfluidic channels with trapezoidal cross-sectional shapes.

The rectangle-shaped tissue chambers used in this paper generated the densest microvascular network. Such results indicate the correlation of the tissue chamber geometry with the densities of the microvasculature formed inside the chambers. In other words, the fluid conditions inside the chambers regulate microvascular development. It has been reported that fluid shear stress attenuates EC sprouting, but the lower shear stress of interstitial flow promotes endothelial morphogenesis and sprout formation^36,37^. Here, in the rectangle-shaped tissue chamber, shear stress was lower than that in chambers with other shapes, which indicates that lower shear stress resulted in more vessel sprouts and denser vascular networks. This finding is in line with the findings in the literature. In conclusion, the correlation between the chamber shape and the resulting vascular density gives us a potential approach to regulate vascular morphogenesis in our modular microfluidic devices according to application needs.

## Conclusion

We developed a modular microfluidic system for large-scale formation of perfused vascularized tissues using a two-layer PDMS configuration. To prevent gel bursting during cell loading at the vertical interface between the layers, a novel plastic mold-based fabrication method was developed to generate semiclosed rhombic medium channels. Microvascular networks with complex morphology and densities were generated using this two-layer, modular microfluidic system. Vertical anastomosis between capillaries in the bottom layer and the medium channels in the upper layer was demonstrated, and 70-kDa FITC-dextran perfusion was demonstrated without nonphysiological leakage. Large-scale microvascular networks covering more than 14 mm^2^ with a vessel density of 12 mm/mm^2^ were generated. This modular microfluidic system provides flexibility and scalability for the development of perfused microvascular networks in vitro, which has high potential for body-on-a-chip applications by connecting multiple organ types in the same platform.

## Supplementary information

Supplementary Materials

Bubble prevention of rhombic medium channel

Microvascular networks at communication pores
