# Supplementary material for: A modular microfluidic system based on a multilayered configuration to generate large-scale perfusable microvascular networks
Source: Microsyst Nanoeng. 2021 Jan 6;7:4. doi: 10.1038/s41378-020-00229-8 (PMC7787972; doi:10.1038/s41378-020-00229-8)
Supplement: Supplementary file 1 — Supplementary Materials [file 41378_2020_229_MOESM1_ESM.docx]

A modular microfluidic system based on a multilayered configuration to generate large-scale perfusable microvascular networks

**Authors:** Tao Yue^1,2 #^, Da Zhao^1 #^, Duc T. T. Phan^3^, Xiaolin Wang^4,5,6^, Joshua Jonghyun Park^7^, Zayn Biviji^8^, Christopher C. W. Hughes^1,3^ and Abraham P. Lee^1,9^*

**Affiliations:**

^1^ Department of Biomedical Engineering, University of California, Irvine, USA

^2^ School of Mechatronic Engineering and Automation, Shanghai University, China

^3^ Department of Molecular Biology and Biochemistry, University of California, Irvine, USA

^4^ Department of Micro/Nano Electronics, Shanghai Jiao Tong University, China

^5^ National Key Laboratory of Science and Technology on Micro/Nano Fabrication, Shanghai Jiao Tong University, China

^6^ Key Laboratory for Thin Film and Micro Fabrication of the Ministry of Education, Shanghai Jiao Tong University, China

^7^ Department of Electrical Engineering and Computer Science, University of California, Irvine, USA

^8^ Department of Applied Mathematics - Biology, Brown University, USA

^9^ Department of Mechanical and Aerospace Engineering, University of California, Irvine, USA

* Correspondence to: Abraham P. Lee (aplee@uci.edu)

^#^ These authors contributed equally to this work.

**Supplementary Materials:**

**1. On-chip culture and perfuse using hydrostatic pressure**


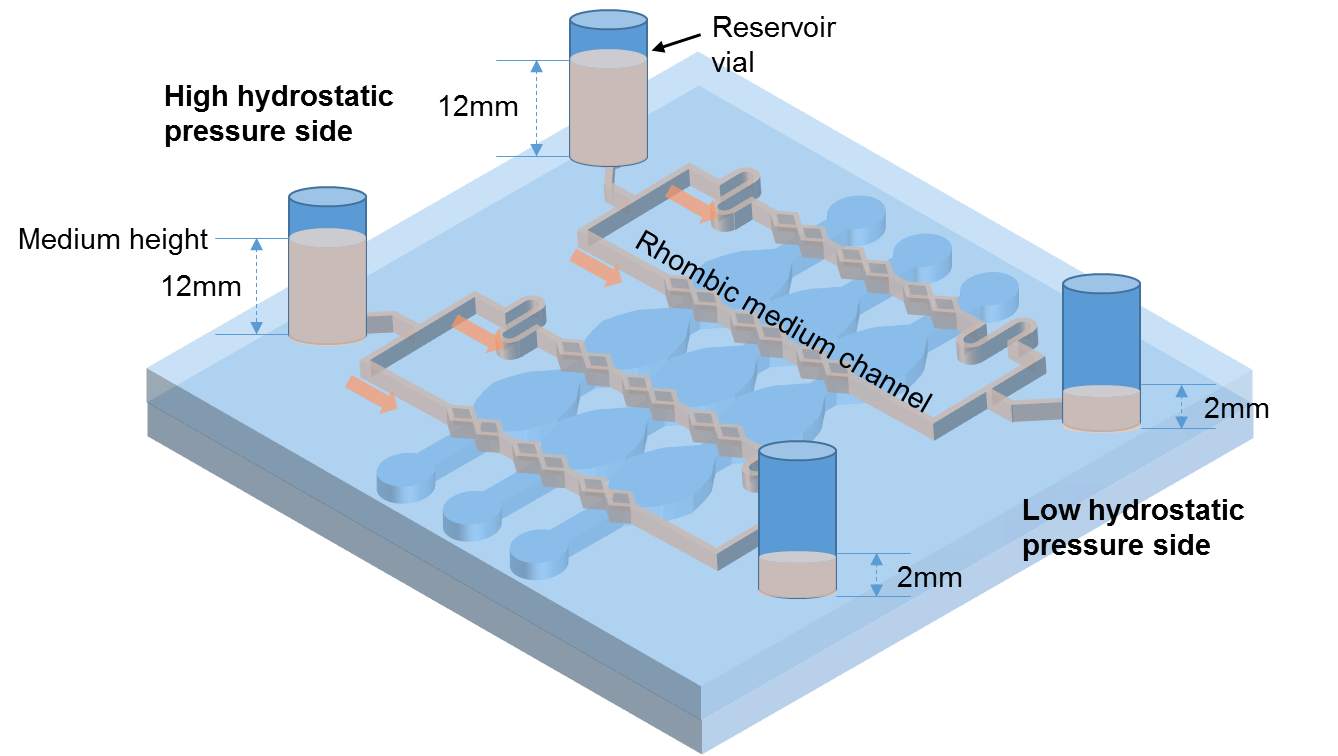


**Figure S1:** *Culture medium in the four reservoir vials has different height to generate hydrostatic pressure. Initially, the medium level at one side of the both rhombic medium channels is the same and is 10 mm higher than the other side.*

**2. Various interstitial flow profiles are provided in the modular microfluidic system**


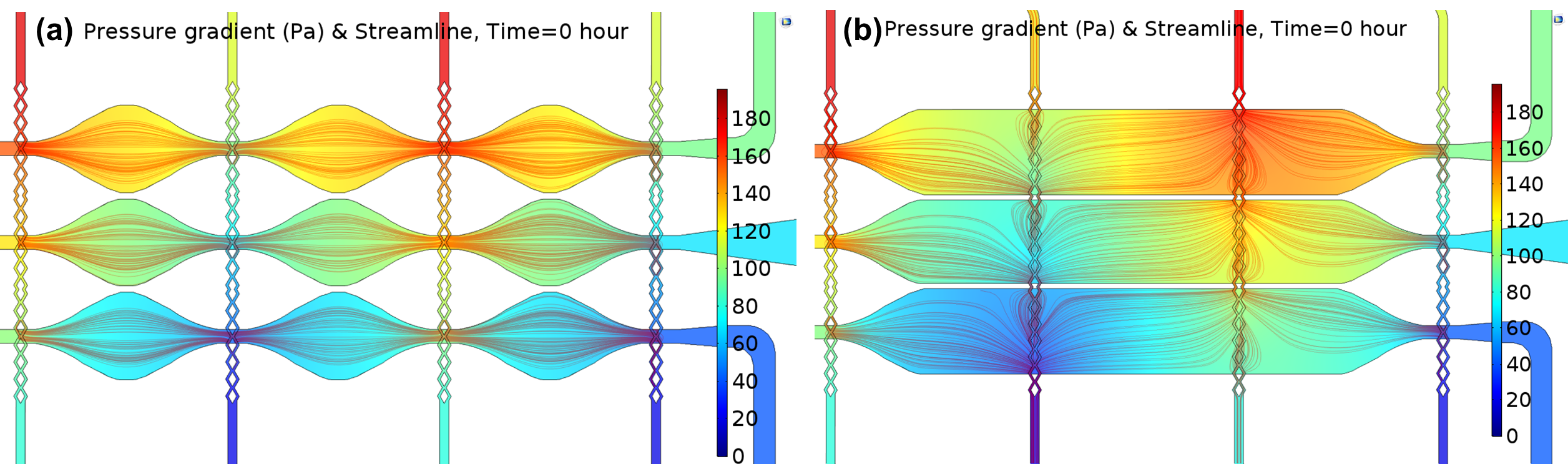


**Figure S2:** *Simulation results of the fluidic conditions inside different tissue chambers. A design for generating relatively low density of capillaries is shown in (a). Another design for large-scale and denser microvascular networks is shown in (b).*

**3. Bubble prevention of rhombic medium channel**

**Video S1** shows the flow inside the rhombic channel when introducing the culture medium without trapping bubbles.


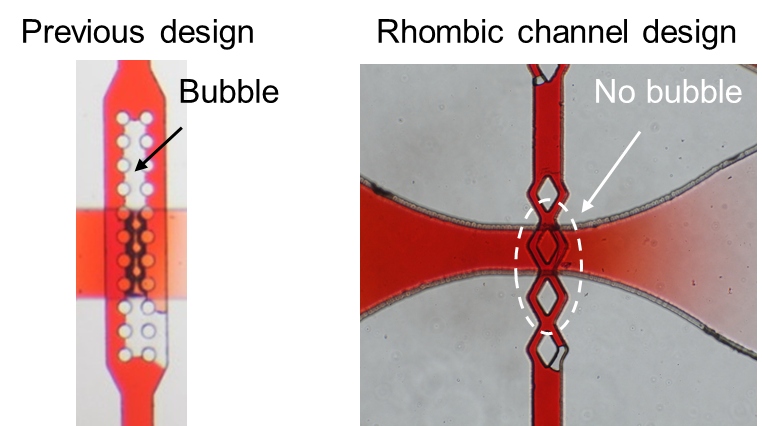


**Figure S3:** *Bubble prevention of the rhombic medium channel.*

**4. Time-lapse images of the microvascular networks development during 11 days**


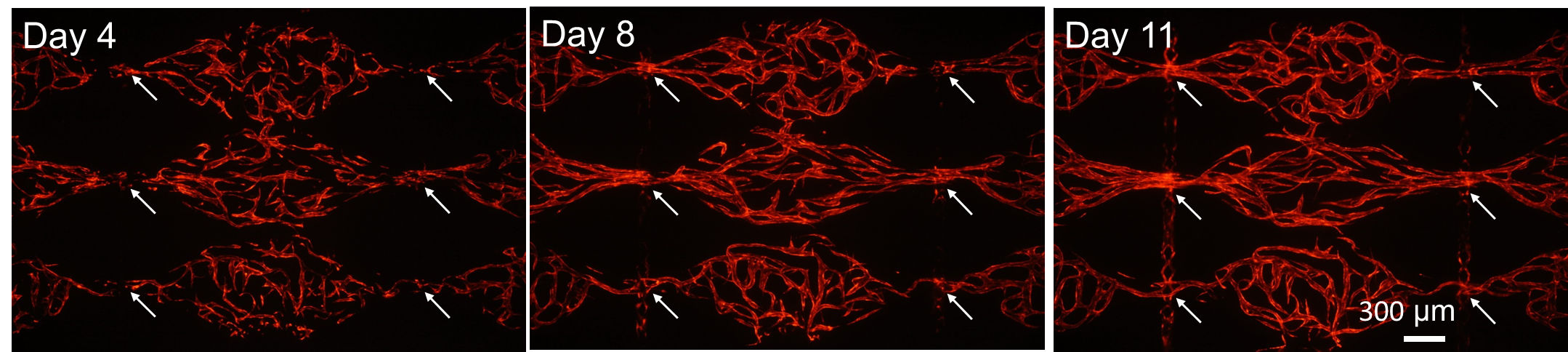


**Figure S4:** *Images showing the growth of capillaries inside the two-layered devices. The arrows point the communication pores where the ECs grow vertically into the medium channels in upper layer, connecting the microvascular networks in bottom layer, which is called vertical anastomosis.*

**5. Confocal images of the vessels confirm the lumens inside as well as the connections between capillaries in bottom layer and channels in upper layer.**


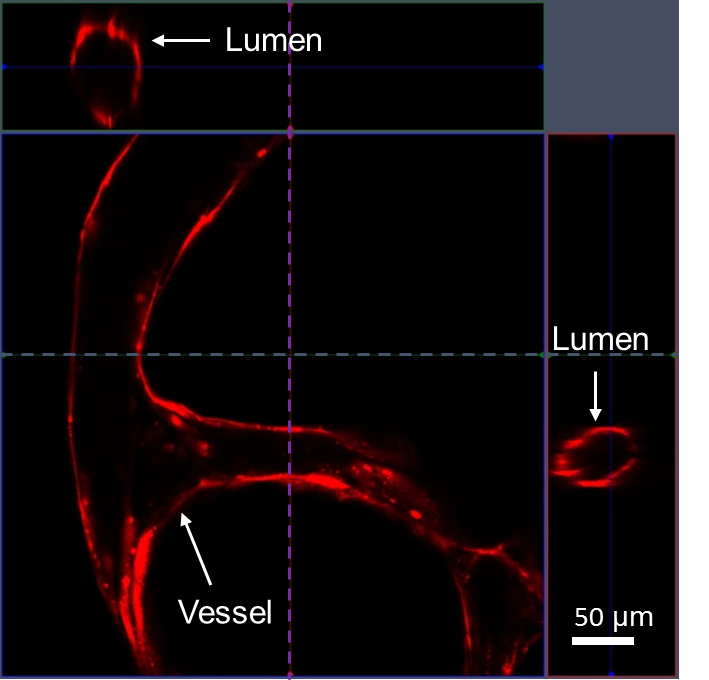


**Figure S5:** *Images showing lumens of the vessels in microvascular networks before doing the perfusing tests.*

**Video S2** shows the complex connections between microvascular networks and medium channels at communication pores, though which the culture medium flows into the capillaries.

**6. Comparison of the vessel density between previous one-layered design and presented two-layered devices**


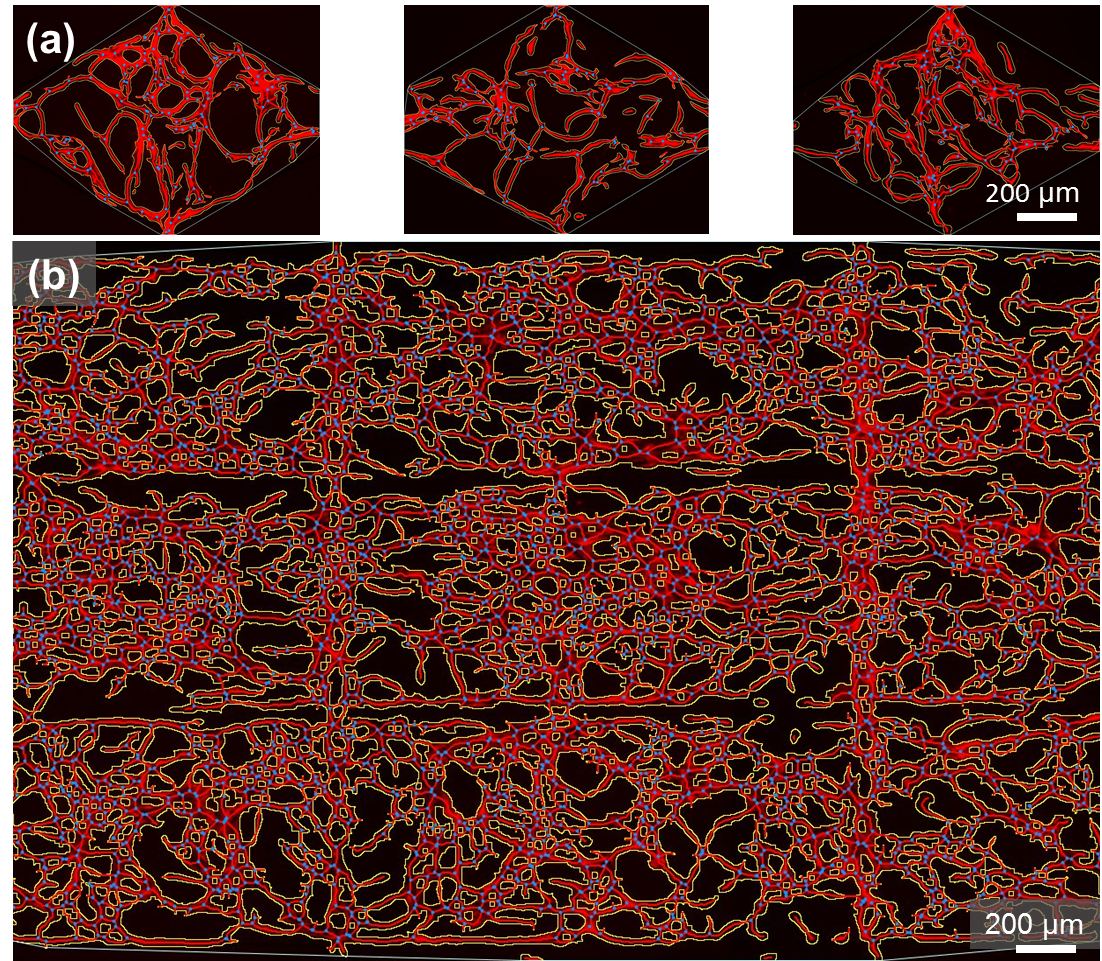


**Figure S6:** *Vessel density increased dramatically, from about 4 mm/mm^2^ in* ***(a)*** *to 12 mm/mm^2^ in* ***(b)****.*

**7. 3D aligning system for multilayered PDMS device assembly**

When assembling the 2 PDMS layers, it’s very difficult to look at the detail of channel features and manipulate the chip in small motions due to the small dimension of microfluidics channels. To address this problem, an aligning system. is built allowing observing the channel feature with magnifying cameras and manipulating the PDMS slabs in 4 degree of freedom (X, Y, Z and 𝜎). The aligning system is made with 2 general parts: cameras and the aligning stage. The cameras are fixed to separated holders that have 3 degree of freedom, so that the cameras can be moved to observe different part of the microfluidics chip for aligning markers. The aligning stage can be divided into two parts. The ceiling glass is held by three point, and two of which can be adjust with different height to make sure the ceiling glass is level. The bottom stage can control the bottom part of the PDMS slab to move in X, Y, Z and 𝜎. During the aligning process, the top part PDMS slab is stuck on to the bottom of the ceiling glass and adjusted to right field of view for the cameras. The bottom PDMS slab is stuck on the bottom stage after the Z position is preadjusted to very close to the top PDMS slab. The position where these two parts are is called the pre-aligned position. Then the bottom PDMS slab and the top PDMS slab with ceiling glass are put the plasma cleaner for plasma treatment. Once the treatment is finished, the two PDMS are put back to pre-aligned position. The ceiling glass is quickly check for level. The bottom PDMS slab is quickly adjusted for X, Y and 𝜎 position for the best aligning effect. At last, the bottom stage is adjusted to rise the Z position to adhere the bottom PDMS slab to the top PDMS slab. The breadboard and the motion control stages are purchase from Newport Corporation. The rest of the holder parts are made by 3D printing in our lab.


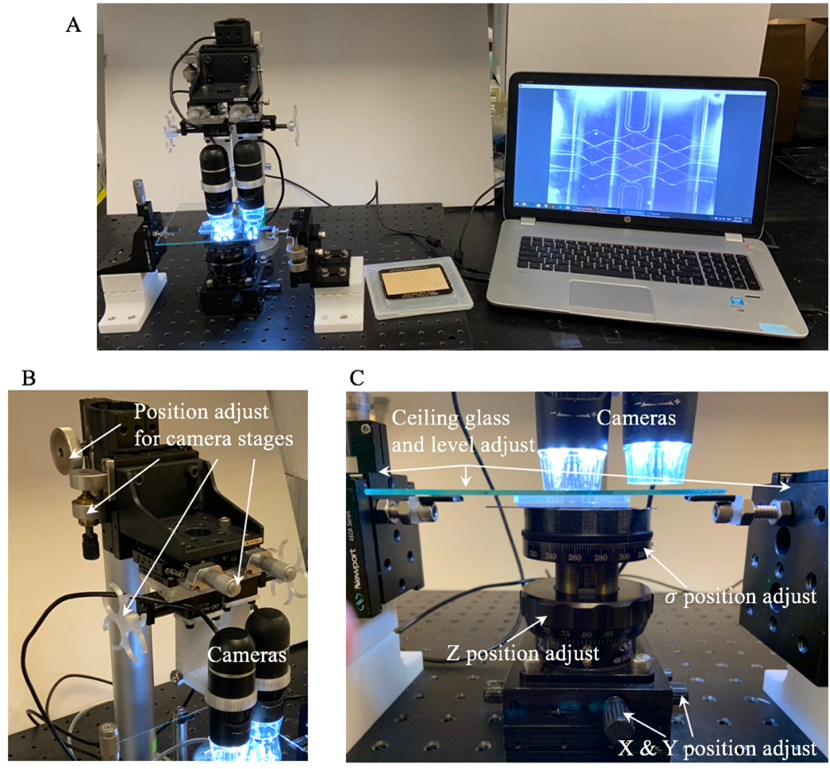


**Figure S7:** *3D aligning system for multilayered PDMS device assembly. (A) The whole system for 3D aligning. A two-layer microfluidics chip described in Chapter 3 is used as a demonstration. (B) The cameras and moving stages. These moving stages allow the cameras to move in X, Y, and Z position, as well as tilt to an angle. (C) Aligning stage. The ceiling glass is held by three points allowing level adjusting. The bottom stage allows the adjusting of X, Y, Z and 𝜎 position for the bottom PDMS slab.*
